# The Relationship between Adjacent Segment Pathology and Facet Joint Violation by Pedicle Screw after Posterior Lumbar Instrumentation Surgery

**DOI:** 10.3390/jcm10132911

**Published:** 2021-06-29

**Authors:** Ho-Seok Oh, Hyoung-Yeon Seo

**Affiliations:** Department of Orthopedic Surgery, Chonnam National University Hospital, Gwangju 61469, Korea; koreankid07@naver.com

**Keywords:** adjacent segment pathology, facet joint violation, posterior lumbar instrumentation, pedicle screw

## Abstract

Transpedicular screw instrumentation systems have been increasingly utilized during the fusion of lumbar spine procedures. The superior segment facet joint violation of the pedicle screw is thought to have potential for accelerating symptomatic adjacent-segment pathology (ASP). The purpose of this study was to investigate the relationship between the superior segment facet joint violation by transpedicular screws and the development of ASP. Among all patients who underwent operations involving one- or two-level posterior lumbar arthrodesis at the Chonnam National University Hospital from 1992 to 2012, 87 patients were selected for this study. Fifty-six patients were included in the ASP group, and 31 were included in the non-ASP group. We used lumbar three-dimensional computed tomography (CT) to assess the violation of the superior facet joint by a transpedicular screw. The assessment is presented in scores ranging from zero to two, with zero indicating no violation (type I); one point indicating suspected violation (type II); and two points indicating definitely facet joint violation (type III). Facet violation was reported in 31 patients in the ASP group (*n* = 56), and in 13 patients in the non-ASP group (*n* = 31). The types of facet joint violation according to our scoring system were as follows: type I, 59 screws (52.7%); type II, 26 screws (23.2%); and type III, 27 screws (24.1%) in the ASP group; and type I, 43 screws (69.4%), type II, 14 screws (22.6 %); and type III, 5 screws (8.0%) in the non-ASP group. The score of facet joint violation in each patient according to our scoring system were as follows: 0 points, 25 patients (44.6%); 1 point, 8 patients (14.3%); 2 points, 4 patients (7.1%); 3 points, 11 patients (19.7%); 4 points, 8 patients (14.3%) in the ASP group; and 0 points, 18 patients (58.1%); 1 point, 4 patients (12.9%); 2 points, 7 patients (22.6%); 3 points, 2 patients (6.4%); 4 points, 0 patients (0%) in the non-ASP group. The mean scores were 1.4 points in the ASP group and 0.8 points in the non-ASP group (*p* < 0.05). We conclude that the position of the pedicle screw farther away from the facet joint surface can reduce the degeneration of the superior adjacent segment. Therefore, close attention to the screw position during surgery may reduce the rate of superior adjacent-segment pathologies.

## 1. Introduction

In recent years, the use of pedicle screw instrumentation systems in the fusion of the lumbar spine has been on the rise [1,2]. Instrumented lumbar fusion is an effective and durable treatment strategy for numerous spinal pathologies, including degenerative lumbar conditions [3]. Implementation of pedicle screw rods has been associated with increased fusion rates and has been shown to facilitate the mobilization of postoperative patients without bracing [4,5,6]. Despite overall clinical success of the technique, associated complications have been reported, such as pedicle violation, neurologic injuries, pseudoarthrosis, instrumentation failures, and facet joint violation [7,8,9,10,11,12,13]. Among these, the superior segment facet joint violation of the pedicle screw could promote symptomatic adjacent-segment pathology (ASP) [2,14,15,16].

Injury to the superior facet joints during placement of pedicle screws causes facet joint stiffness, rigidity, and osteoarthritis. Moreover, facet joint violation may contribute to increasing stress at the adjacent segment, leading to biomechanical changes including abnormal facet joint loading and aberrant motion, which can accelerate ASP [17]. Abnormal loading and increased mobility in adjacent segments may explain the mechanism behind the development of ASP; however, it is unclear whether it is caused by fusion sequelae or whether it is the result of natural degeneration. Moreover, the exact incidence of ASP and its risk factors are a subject of controversy [18].

The purpose of this study was to investigate the relationship between superior segment facet joint violation by transpedicular screws and the development of ASP. Following, we aimed at predicting the risk of ASP through quantitative evaluation of facet joint violation.

## 2. Materials and Methods

In this study, we enrolled patients who had undergone L4/5, L5/S1, or L4/5/S1 posterior lumbar interbody fusion (PLIF) or posterolateral fusion (PLF) for a degenerative disease at the Chonnam National University Hospital (CHUH) from 1992 to 2012. Criteria for inclusion in our study were: age older than 18 years, bilateral pedicle screw fixation for fusion of the thoracolumbar spine, midline surgical approach, and CT scan within 1 year of the surgical procedure. In addition, patients with a history of lumbar surgery or trauma were excluded.

Adjacent segmental pathology (ASP) was diagnosed when plain radiographs, computed tomography (CT), or magnetic resonance imaging (MRI) demonstrated one or more of the following lesions (absent preoperatively) at the segment adjacent to the fused segment: more than 4 mm of anterolisthesis or retrolisthesis, more than 10° of angular motion between adjacent vertebral bodies, more than 50% loss of disc height, or more than 1 grade advancement of facet joint degeneration or disc degeneration or spinal canal stenosis [18].

Asymptomatic patients without disc-space narrowing, segmental instability, or facet arthropathy shown on a follow-up radiograph were defined as non-ASP patients. Finally, 87 patients met all the inclusion criteria of this study. Among the 87 patients, 56 patients were included in the ASP group, and 31 patients were allocated to the non-ASP group.

**Figure 1:**
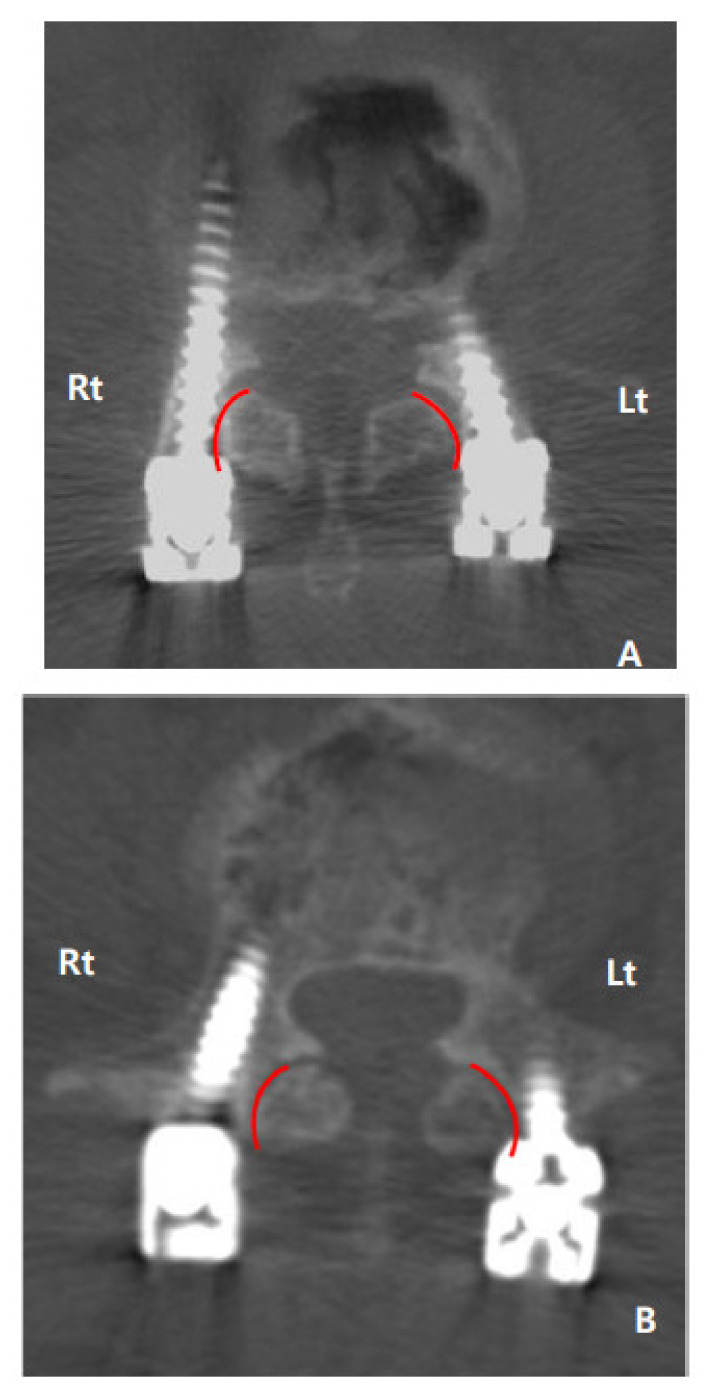
Superior facet joint violation by transpedicular screw evaluated using computed tomography. (**A**) The right is 2 points, the left is 1 point, and the final score is 3 points; (**B**) the right is 0 point, the left is 2 points, and the final score is 2 points.

Lumbar 3D CT was performed to assess superior facet joint violation caused by transpedicular screws. All the CT results were thoroughly assessed by two experienced spine orthopedists. Two evaluators were trained in advance to gain sufficient expertise in superior facet joint violation. No clinical history or patient information was known by the evaluators during the evaluations. The evaluated outcomes are presented as a score measure, i.e., 0, non-violated (type I); 1, suspected violation (type II); 2, definite violation (type III) (Figure 1). After scoring each of the superior facet joint violation on both sides, the sum of both sides was determined as the final score.

### Statistical Analysis

All statistical analyses were performed using the Statistical Package for Social Sciences software, version 22.0 (SPSS, Inc., Chicago, IL, USA). Continuous variables were measured as mean ± standard deviation, and categorical variables were expressed as frequencies or percentage. To assess the significance of intergroup differences, an independent *t*-test was used for age, follow-up duration, and scores. Chi-square tests were used for categorical variables, such as sex, preoperative diagnosis, operative level, and violated pedicle level. Values of *p* < 0.05 were considered to be statistically significant. All analyses were independently reviewed by a statistician. The kappa coefficient was calculated to test the inter-rater reliability of evaluating the violation caused by transpedicular screws. Cohen’s kappa value of 0.61 to 0.80 was considered to be a substantial agreement and from 0.81 to 1.00 was considered to be an almost perfect agreement.

## 3. Results

Twenty-six men and 30 women were included in the ASP group (mean age 64.8 years, range 34.4–75.5), and a mean follow-up duration was 13.7 years (range 2.2–23.6). Fourteen men and 17 women were included in the non-ASP group (mean age 57.5 years, range 38.4–75.8), and a mean follow-up duration was 11.7 years (range 2.3–22.0). In the ASP group, 33 patients were preoperatively diagnosed with spinal stenosis and 23 patients with spondylolisthesis. In the non-ASP group, 15 and 16 patients were diagnosed with spinal stenosis and spondylolisthesis, respectively. There were 35 cases of L4-5, 7 cases of L5-S1, and 14 cases of L4-5-S1 in the ASP group, and there were 20, 4, and 7 cases of L4-5, L5-S1, and L4-5-S1, respectively, in the non-ASP group. The average time from surgery to diagnosis of ASP was 3.2 years (range 0.9–5.4). The pre-operative demographic data did not differ significantly between the groups (Table 1).

Facet violation was seen in 53 (47.3%) of 112 screws in the ASP group, and 19 (30.6%) of 62 screws in the non-ASP group. Among them, 13 patients (26 screws, 23.2%) in the ASP group and 3 patients (6 screws, 9.7%) in the non-ASP group showed bilateral facet joint violation, accounting for a significant difference between the two groups.

Forty-four (44.9%) of the 98 screws inserted in the L4 in the ASP group and 9 (64.3%) of the 14 screws inserted in the L5 in the ASP group were associated with facet joint violation. In the non-ASP group, 17 (31.5%) of the 54 screws in the L4 and 2 (25%) of 8 screws in the L5 showed facet joint violation, and there were no statistically significant differences between the two groups. In patients diagnosed with spinal stenosis, 32 (48.5%) of 66 screws in the ASP group and 11 (36.7%) of 30 screws in the non-ASP group were observed. In patients diagnosed with spondylolisthesis, 21 (45.7%) of 46 screws in the ASP group and 8 (25.0%) of 32 screws in the non-ASP group were observed. No statistically significant differences between the two groups in facet joint violation by preoperative diagnosis were detected (Table 2).

According to our scoring system, the facet joint violation types were as follows: type I, 59 screws (52.7 %); type II, 26 screws (23.2 %); and type III, 27 screws (24.1 %) in the ASP group, and type I, 43 screws (69.4 %); type II, 14 screws (22.6 %); and type III, 5 screws (8.0 %) in the non-ASP group (Table 3). The score of facet joint violation in each patient according to our scoring system were as follows: 0 points, 25 patients (44.6%); 1 point, 8 patients (14.3%); 2 points, 4 patients (7.1%); 3 points, 11 patients (19.7%); 4 points, 8 patients (14.3%) in the ASP group; and 0 points, 18 patients (58.1%); 1 point, 4 patients (12.9%); 2 points, 7 patients (22.6%); 3 points, 2 patients (6.4%); 4 points, 0 patients (0%) in the non-ASP group. The mean scores were 1.4 points in the ASP group and 0.8 points in the non-ASP group (*p* < 0.05) (Table 4). The inter observer kappa coefficient was 0.93 (i.e., almost perfect).

## 4. Discussion

Lumbar fusion surgery constitutes a widely accepted treatment strategy for lumbar diseases, such as lumbar stenosis, trauma, tumors, and spondylolisthesis. Development of new instruments and improved bone graft materials have resulted in higher fusion and clinical success rates. Nevertheless, many complications and problems related to fusion surgery have been reported, with ASP being one of the most troublesome issues [18,19,20].

The various types of pathological changes of the adjacent segments are spondylolisthesis, canal stenosis, disc herniation, disc height loss, osteophyte formation, and scoliosis. The incidence of radiographic adjacent segmental pathology ranges from 8% to as high as 100%, with a symptomatic disease in 5.2%–18.5% of cases, as reported in studies with an average follow-up of 36–396 months [21]. The most common reported risk factors for developing ASP are age, sex, body mass index (BMI), smoking, osteoporosis, preexisting degeneration of an adjacent disc, violation of adjacent facet during surgery, sagittal imbalance, rigid fixation, and length of fusion [21]. Despite extensive research, the exact causes of ASP are not fully elucidated, but changes in spinal biomechanics, including increased facet loading, increased intradiscal pressure, and hypermobility at the segments adjacent to the fusion levels, are thought to play a key role is ASP pathogenesis [21]. Weinhoffer et al. found that as flexion motion increases, intradiscal pressure increases within adjacent levels [22]. Umehara et al. reported a significant increase in weight bearing and load burden of the posterior column at the adjacent segments following lumbar fusion [23].

Shah et al. reported that facet joint violation occurred in over 30% of patients and was triggered by 20% of screws [13]. Moshifar et al. reported that top-level facet joint violations occurred in 15% of cases with cephalad pedicle screws and in 24% of patients [24]. In our study, as mentioned above, 55.4% of the cases and 47.3% of the screws in the ASP group and 41.9% of the cases and 30.6% of the screws in the non-ASP group were associated with facet joint violations. The incidence of facet joint violation reported here was higher than other studies, as we applied stricter diagnostic criteria, for example, by including any doubtful part in C.T.

Cardoso et al. reported that bilateral facet joint violation contributes to torsional instability after surgery [6]. In our study, 13 patients with 26 screws (23.2%) in the ASP group and 3 patients with 6 screws in the non-ASP group (9.7%) showed signs of bilateral facet joint violation, accounting for a significant difference between the groups (*p* < 0.5). This indicated that bilateral facet joint violation plays a role in the onset of ASP.

Moshifar et al. reported that when the cephalad pedicle screws were at the L5 (L5-S1 fusion), facet joint violations were significantly more likely to occur as compared with any other level [24]. In our study, since the L3-4 facet joint is the most superior facet (L4-5 and L4-5-S1 fusion), there was no difference between 44 of 98 screws (44.9%) in the ASP group and 17 of 54 screws (31.5%) in the non-ASP group. However, since the L4-5 facet joint is the most superior facet joint (L5-S1 fusion), 9 of 14 screws (64.3%) in the ASP group and 2 of 8 screws (25%) in the non-ASP group showed facet joint violation. A study on a higher number of patients with superior segment facet joint violations will be necessary to evaluate the significance of these findings.

The most important finding of the current study is that the ASP group showed higher superior facet joint violation rates than the non-ASP group: 31 patients (55.4%) had 53 screws (47.3%) which violated the superior facet joint in the ASP group and 13 patients (41.9%) had 19 screws (30.6%) which violated the superior facet joint in the non-ASP group (*p* < 0.05). Additionally, the bilateral facet joint violation rate was higher in the ASP group than in the non-ASP group: 13 patients (23.2%) had 26 screws (23.2%) which violated the superior facet joint in the ASP group, and 3 patients (9.7%) had 6 screws (9.7%) which violated the superior facet joint in the non-ASP group (*p* < 0.05). In addition, facet joint involvement was scored according to its severity; the ASP group had an average score of 1.4, whereas the non-ASP group had a score of 0.8 (*p* < 0.05). These results point to superior facet joint violation caused by transpedicular screws as an important factor in the development of ASP in this study. However, according to result of this study, ASP also occurred in 25 patients without facet joint violation. This is probably due to the presence of other important factors that cause ASP in addition to facet joint violation, and it is necessary to identify other factors that affect ASP thorough further research.

There are some limitations of our study. First, the number of patients in this study is small. If the follow-up duration was longer, it seems that clearer results could be obtained. Second, as mentioned above, in addition to facet violation, there are many other important factors that affect ASP including length of fusion, type of instrumentation, sagittal parameters, etc. How these factors actually affect ASP needs to be clarified through future study. Third, as with any retrospective study, selection biases may be present.

## 5. Conclusions

We detected significant differences in the facet joint violation after transpedicular screw insertion between the ASP group and the non-ASP group in this retrospective study. In the pedicle screw fixation for a degenerative lumbar disease, the superior segment facet joint violation by the pedicle screw increases the incidence of upper ASP. The position of the pedicle screw farther away from the facet joint surface can reduce the degeneration of the superior adjacent segment. Therefore, careful attention during surgical procedures may reduce the incidence of superior adjacent-segment pathology.

## Figures and Tables

**Table 1:** Preoperative demographic data.

	ASP Group (*n* = 56)	Non-ASP Group (*n* = 31)	*p*-Value
Sex, male/female (n)	26/30	14/17	0.91
Age (y)	64.8 ± 7.01	57.5 ± 8.76	0.38
BMI (Kg/m^2^)	24.75 ± 2.93	24.93 ± 2.30	0.77
Follow up duration (y)	13.7 ± 5.73	11.7 ± 6.26	0.13
Preoperative diagnosis (n)			
Spinal stenosis	33	15	0.34
Spondylolisthesis	23	16	
Level of operation (n)			
L4-5	35	20	
L5-S1	7	4	
L4-5-S1	14	7	

Pearson’s chi-square test, independent *t*-test data are presented as median ± standard deviation. The *p*-values are of inter-group comparisons, with *p* < 0.05 indicating statistical significance.

**Table 2:** Distribution of superior facet joint violations by patient clinical characteristics in the ASP group and non-ASP group.

	ASP Group (*n* = 56, 112 screws)	Non-ASP Group (*n* = 31, 62 Screws)	*p*-Value
Facet violation	53 screws (47.3%, *n* = 40)	19 screws (30.6%, *n* = 16)	<0.05
Bilateral violation	26 screws (23.2%, *n* = 13)	6 screws (9.7%, *n* = 3)	<0.05
Violation level			
L4	44 screws (of 98 screws, 44.9%)	17 screws (of 54 screws, 31.5%)	0.72
L5	9 screws (of 14 screws, 64.3%)	2 screws (of 8 screws, 25%)	
Diagnosis			
Spinal stenosis	32 screws (of 66 screws, 48.5%)	11 screws (of 30 screws, 36.7%)	0.53
Spondylolisthesis	21 screws (of 46 screws, 45.7%)	8 screws (of 32 screws, 25%)	

Pearson’s chi-square test. The *p*-values are of inter-group comparisons, with *p* < 0.05 indicating statistical significance.

**Table 3:** Incidence of superior facet joint violation on CT scan in the ASP group and non-ASP group.

Type (Score)	ASP Group (112 Screws)	Non-ASP Group (62 Screws)
I (0)	59 screws (52.7%)	43 screws (69.4%)
II (1)	26 screws (23.2%)	14 screws (22.6%)
III (2)	27 screws (24.1%)	5 screws (8.0%)

Non-violated (Type I); if violation is suspected (type II); facet joint is definitely violated by the screw (type III).

**Table 4:** Score of facet joint violation.

		ASP Group (*n* = 56)	Non-ASP Group (*n* = 31)	*p*-Value
Score	0	25 (45%)	18 (58%)	
	1	8 (14%)	4 (13%)	
	2	4 (7%)	7 (23%)	
	3	11 (20%)	2 (6%)	
	4	8 (14%)	0 (0%)	
	Mean score	1.4	0.8	<0.05

Score measures: 0, if non-violated (Type I); 1, if violation is suspected (type II); 2, if facet joint is definitely violated by the screw (type III). Independent *t*-test data are presented as median ± standard deviation. The *p*-values are of inter-group comparisons, with *p* < 0.05 indicating statistical significance.

## Data Availability

The data presented in this study are available on request from the corresponding author.
